# Family-centered mental health care for left-behind children in Chinese ethnic minority concentrated areas: a psychiatric nursing framework for rural education settings

**DOI:** 10.3389/fpubh.2025.1709606

**Published:** 2025-12-11

**Authors:** Yi Rong, Guangzhe Frank Yuan

**Affiliations:** 1 School of Education Science, Leshan Normal Univeristy, Leshan, China; 2 Sichuan Rural Education Development Research Center, Leshan Normal University, Leshan, China

**Keywords:** family-centered care, left-behind children, ethnic minority, psychiatric nursing, mental health disparities

## Abstract

**Background:**

Left-behind children in Chinese ethnic minority concentrated areas face significant mental health challenges stemming from parental migration, cultural factors, and limited access to care. To address this, culturally appropriate, family-centered interventions are required.

**Purpose:**

This paper proposes a comprehensive psychiatric nursing framework to deliver culturally responsive, family-centered mental health care within rural educational settings serving ethnic minority concentrated areas in China.

**Methods:**

This framework development study integrates evidence from comprehensive literature synthesis across multiple domains: family-centered care, cultural competence, rural mental health disparities, school-based service delivery, and psychiatric nursing practice. The framework is grounded in three complementary theoretical perspectives: Family Stress Model, Cultural Competence Theory, and Ecological Systems Theory. Development involved iterative synthesis of empirical evidence, theoretical integration, and incorporation of implementation science principles.

**Framework components:**

The proposed framework encompasses four core components: (1) culturally responsive assessment protocols incorporating multi-informant approaches and culturally adapted screening instruments; (2) family-centered intervention strategies including multi-generational family therapy, communication enhancement, and cultural identity development; (3) school-based mental health service delivery featuring integrated services, teacher training, and peer support programs; and (4) community partnership models engaging health care systems, cultural institutions, and community advisory boards. Implementation occurs in three phases: Preparation and Planning, Pilot Implementation, and Full Implementation and Sustainability. A comprehensive evaluation framework examines both process indicators (fidelity, utilization, stakeholder satisfaction) and outcomes (child mental health, family functioning, educational performance, community capacity).

**Implementation and evaluation:**

The framework includes detailed implementation strategies across three phases, comprehensive training and capacity-building protocols for implementation teams, and a dual-focus evaluation framework examining both process measures and multi-level outcomes.

**Conclusion:**

Implementing this framework can reduce mental health disparities among left-behind children in ethnic minority rural areas by strengthening family resilience and community support. Integrating these services within schools offers a sustainable and accessible care delivery model for this underserved population.

## 1 Introduction

China’s rapid economic development and unprecedented urbanization have created a massive internal migration phenomenon, resulting in approximately 67 million left-behind children separated from their migrant parents (1). The term “left-behind children” refers to children under 18 years of age who remain in their rural home communities while one or both parents migrate to urban areas for work, typically for extended periods (months to years). These children are usually cared for by grandparents, other extended family members, or in some cases, left to care for themselves or younger siblings. This phenomenon is particularly prevalent in China due to the hukou (household registration) system, which restricts rural migrants’ access to education and social services in urban areas, making it impractical for families to relocate together. While most common in China, similar patterns of parental labor migration leaving children behind exist in other developing countries experiencing rapid urbanization and rural-to-urban migration, including parts of Southeast Asia, Latin America, and Sub-Saharan Africa. Understanding this phenomenon has important implications for child development, family systems, and mental health service delivery in any context where economic pressures drive parental migration and family separation. Among these vulnerable populations, children in ethnic minority concentrated areas face particularly complex challenges, experiencing higher rates of mental health problems compared to their non-minority counterparts (2). The intersection of parental absence, cultural identity challenges, rural isolation, and limited mental health resources creates a perfect storm of risk factors that demand innovative, culturally responsive interventions.

Left-behind children in Chinese ethnic minority regions demonstrate significantly elevated rates of depression, anxiety, and behavioral problems (3, 4). Research indicates that these children are more prone to experiencing depression and other adverse emotions compared to their urban counterparts, with parental migration patterns significantly affecting their psychological well-being (2). The prevalence of mental health challenges is particularly pronounced when mothers migrate for work, disrupting traditional caregiving structures that are culturally significant in ethnic minority communities.

The current mental health service delivery system in rural China faces substantial challenges in addressing the needs of ethnic minority left-behind children. Rural mental health disparities are well-documented globally, with rural residents experiencing significant barriers to accessing quality mental health care (5). These disparities are compounded in ethnic minority communities by cultural stigma, language barriers, and lack of culturally competent providers (6). Traditional mental health service models, developed primarily for urban populations, fail to address the unique cultural, linguistic, and structural barriers faced by ethnic minority families in rural settings.

Family-centered care has emerged as a promising approach for addressing mental health needs of vulnerable children populations. Research demonstrates that family caregiving significantly improves mental health outcomes for individuals with various mental health conditions, with different methods of enhancing family support showing consistent positive results ((7, 8)). Barnes et al. (9) emphasized that families are crucial actors within public health frameworks, capable of fostering higher capacity for community-level interventions. This approach is particularly relevant for Chinese ethnic minority communities, where extended family networks and intergenerational relationships play central roles in child development and well-being.

Schools represent ideal venues for mental health service delivery in rural areas, serving as primary community institutions with direct access to children and families. Research indicates that schools play an integral role in supporting students’ mental health by providing access to programs, identifying students needing additional support, and linking families to services (10, 11). However, implementation of school-based mental health supports varies significantly, with rural schools and those serving high-poverty populations less likely to implement comprehensive mental health programming (12).

Psychiatric nursing offers unique advantages for addressing mental health needs of left-behind children in ethnic minority rural areas. Psychiatric nurses are trained to provide holistic, family-centered care that addresses both individual and systemic factors affecting mental health (13). Their scope of practice includes assessment, intervention, education, and advocacy - skills essential for working with complex, multi-faceted challenges faced by left-behind children and their families. Furthermore, psychiatric nurses can serve as bridge-builders between health care systems, educational institutions, and community organizations, facilitating integrated service delivery models.

Despite growing recognition of these challenges, there remains a significant gap in evidence-based frameworks specifically designed for psychiatric nursing practice with left-behind children in ethnic minority concentrated areas. Existing interventions often lack cultural specificity, fail to address unique family dynamics in ethnic minority communities, ordo not adequately integrate with rural educational systems. This manuscript addresses this critical gap by proposing a comprehensive family-centered psychiatric nursing framework specifically designed for rural education settings serving Chinese ethnic minority concentrated areas.

The proposed framework is grounded in several theoretical foundations: the Family Stress Model (14), which examines how economic hardship and parental absence affect family functioning and child outcomes; Cultural Competence Theory (15), which emphasizes the importance of understanding and respecting cultural values, beliefs, and practices in service delivery; and Ecological Systems Theory (16), which recognizes the multiple levels of influence on child development, from individual and family factors to community and societal influences.

## 2 Literature review

### 2.1 Left-behind children and mental health outcomes

The phenomenon of left-behind children has garnered significant attention in both academic and policy circles. Research consistently demonstrates that parental absence has significant adverse impacts on children’s educational and mental health outcomes (17). A comprehensive analysis reveals that left-behind children have lower cognitive test scores, academic achievement, and are less likely to pursue higher education compared to children living with both parents. These effects vary across gender, parents’ education level, and urban versus rural residence, with more significant impacts observed among girls, students with less-educated parents, and urban students.

The mental health implications of parental separation are particularly severe. Studies indicate that left-behind children experience higher rates of depression, loneliness, and behavioral problems compared to their peers [e.g., Hu et al. (18) and Huang et al. (19)]. Xiong et al. (20) meta-analysis examining loneliness among left-behind children found significantly elevated levels of loneliness, which served as a mediating factor for other mental health problems. The psychological impact extends beyond immediate symptoms, affecting long-term developmental trajectories and social functioning.

Ethnic minority left-behind children face additional challenges that compound these mental health risks. Research examining rural ethnic minority children found that parental migration patterns significantly predict depression subgroups, with children whose mothers migrate for work being particularly vulnerable to developing severe somatic symptoms and diverse depression patterns (2). The intersection of ethnic minority status and left-behind status creates unique vulnerabilities that require specialized intervention approaches.

### 2.2 Rural mental health disparities

Rural populations worldwide experience significant mental health disparities, with rural residents showing higher rates of suicide, substance abuse, and untreated mental health conditions compared to urban populations (5). These disparities are attributed to multiple factors including provider shortages, geographic isolation, transportation barriers, and cultural stigma associated with mental health help-seeking. In rural areas, as many as 65% of nonmetropolitan counties lack psychiatrists, and over 60% of rural Americans live in designated mental health provider shortage areas.

The impact of rural mental health disparities is particularly pronounced for children and adolescents. Rural children from small communities are more likely to have mental, behavioral, and developmental disorders than children living in cities and suburbs, yet access to specialized pediatric mental health services is severely limited (5). This disparity is exacerbated in ethnic minority rural communities, where cultural barriers, language differences, and historical mistrust of formal health care systems further impede access to appropriate care.

School-based mental health services represent a promising strategy for addressing rural mental health disparities. However, research indicates significant variation in implementation of school-based mental health supports, with rural schools less likely to offer comprehensive programming (12). Rural schools face unique challenges including limited funding, staffing shortages, and geographic isolation that impede their ability to implement and maintain mental health programming.

### 2.3 Family-centered care in mental health

Family-centered care has gained recognition as an evidence-based approach for improving mental health outcomes across diverse populations. Research demonstrates that family caregiving interventions consistently improve outcomes for individuals with mental health conditions [e.g., Weinbrecht et al. (21)]. Meta-analyses of family-based interventions show significant improvements in symptom management, treatment adherence, and overall quality of life for both patients and family members [e.g., Hu et al. (7)].

The effectiveness of family-centered care is particularly relevant for Chinese cultural contexts, where family relationships and intergenerational support systems play central roles in child development and well-being. Studies examining family environments in rural China demonstrate significant associations between family communication patterns, caregiver relationships, and children’s psychological well-being [e.g., Liu et al. (22) and Zhou et al. (23)]. Research indicates that positive family environments serve as protective factors against mental health problems, while family dysfunction and poor communication patterns increase risk for depression and behavioral problems.

However, implementation of family-centered care faces unique challenges in ethnic minority rural communities. Traditional family structures may be disrupted by migration patterns, economic pressures, and cultural changes. Many left-behind children are cared for by grandparents or other extended family members who may lack mental health literacy or resources to provide optimal support (24). These caregivers often have limited education, restricted access to information, and may hold traditional beliefs about mental health that conflict with evidence-based treatment approaches.

### 2.4 Psychiatric nursing and cultural competence

Psychiatric nursing offers unique advantages for delivering culturally competent mental health care to ethnic minority populations. Psychiatric nurses are trained to provide holistic care that addresses biological, psychological, social, and cultural factors affecting mental health (13). Their scope of practice includes assessment, diagnosis, treatment planning, intervention delivery, and care coordination - skills essential for addressing complex, multi-faceted needs of left-behind children and their families.

Cultural competence in psychiatric nursing requires understanding and respecting cultural values, beliefs, and practices while delivering evidence-based care. Research examining mental health care for ethnic minority populations emphasizes the importance of cultural adaptation of interventions, provider cultural competence training, and community engagement strategies (6). Effective cultural competence involves not only language translation but also adaptation of assessment tools, intervention strategies, and service delivery models to align with cultural norms and preferences.

Studies examining psychiatric nursing interventions for ethnic minority children demonstrate positive outcomes when cultural competence principles are systematically integrated into practice. Key elements include: (1) cultural assessment protocols that examine family structures, communication patterns, and help-seeking behaviors; (2) culturally adapted screening and assessment tools; (3) intervention strategies that incorporate cultural strengths and resources; and (4) community partnership models that engage traditional healers, religious leaders, and other cultural authorities.

### 2.5 School-based mental health services

Schools represent ideal venues for mental health service delivery, particularly in rural areas where schools often serve as primary community institutions. Research demonstrates that schools play integral roles in supporting students’ mental health by providing access to programs, identifying students in need, and connecting families to services (12). School-based mental health services offer several advantages including: (1) reduced stigma associated with seeking help; (2) improved access through elimination of transportation barriers; (3) integration with educational programming; and (4) opportunities for early identification and intervention.

However, implementation of school-based mental health services faces significant challenges, particularly in rural areas. Research examining implementation of school-based mental health supports found substantial disparities by school characteristics, with rural schools and those serving high-poverty populations less likely to implement comprehensive programming (12). Common barriers include funding limitations, staffing shortages, lack of training and technical assistance, and competing educational priorities.

Successful school-based mental health programs require systematic planning, stakeholder engagement, and ongoing support. Research identifies key implementation factors including: (1) administrative support and leadership commitment; (2) staff training and capacity building; (3) integration with existing school programming; (4) family and community engagement; and (5) sustainable funding mechanisms. Programs that address these implementation factors demonstrate better outcomes and greater sustainability over time.

## 3 Methodology

This article presents a conceptual framework for family-centered psychiatric nursing care developed through systematic synthesis of existing evidence and theoretical integration. This section attempts to describe the methodological approach used to develop the proposed framework.

### 3.1 Framework development approach

This framework was developed using a theory-informed, evidence-based approach consistent with established methods for healthcare framework development (25, 26). The development process involved four iterative stages: (1) comprehensive literature synthesis, (2) theoretical integration, (3) framework component specification, and (4) implementation strategy development. Framework development was guided by principles from implementation science, which emphasizes the importance of designing interventions with explicit attention to contextual factors, stakeholder needs, and feasibility of real-world implementation (25). This approach recognizes that effective frameworks must be both evidence-based and practically implementable within the constraints of rural, resource-limited settings.

### 3.2 Literature synthesis

A comprehensive literature review was conducted across five primary domains relevant to the framework: (1) mental health outcomes and risk factors for left-behind children, particularly in ethnic minority contexts; (2) rural mental health service delivery and disparities; (3) family-centered care approaches in mental health; (4) cultural competence in psychiatric nursing practice; and (5) school-based mental health service implementation.

Literature sources included peer-reviewed empirical research, systematic reviews and meta-analyses, theoretical and conceptual papers, implementation studies, and policy documents. Electronic databases searched included PubMed, CINAHL, PsycINFO, and Chinese databases (CNKI, Wanfang) to ensure inclusion of relevant Chinese-language literature. Search terms combined concepts related to left-behind children, ethnic minorities, mental health, family-centered care, cultural competence, school-based services, and psychiatric nursing. Inclusion criteria for literature synthesis were: (1) relevance to one or more framework domains; (2) focus on child/adolescent populations, particularly vulnerable or underserved groups; (3) empirical research, systematic reviews, or well-grounded theoretical/conceptual work; and (4) applicability to rural, low-resource, or ethnic minority contexts. Literature was synthesized thematically to identify: evidence-based practices and interventions; theoretical frameworks and models; implementation strategies and barriers; cultural considerations; and gaps in existing knowledge and practice.

### 3.3 Theoretical integration

Following literature synthesis, relevant theoretical frameworks were identified and integrated to provide conceptual foundations for the proposed framework. Three complementary theories were selected based on their relevance to understanding the multi-level challenges faced by left-behind children in ethnic minority rural areas: the Family Stress Model (14), Cultural Competence Theory (15), and Ecological Systems Theory (16). These theoretical perspectives were integrated to create a comprehensive understanding of: (1) how economic hardship and family disruption affect child outcomes (Family Stress Model); (2) how cultural factors influence mental health, help-seeking, and intervention effectiveness (Cultural Competence Theory); and (3) how multiple system levels (individual, family, school, community, society) interact to influence child development (Ecological Systems Theory). This theoretical integration ensured that framework components address individual, family, cultural, and systemic factors simultaneously.

### 3.4 Framework component specification

Based on the integrated evidence and theoretical foundations, specific framework components were delineated. The framework structure was organized around four core components, each addressing a different aspect of comprehensive mental health service delivery: (1) assessment protocols, (2) intervention strategies, (3) service delivery mechanisms, and (4) community partnerships. For each component, specific elements were specified including: evidence-based practices and strategies drawn from the literature; cultural adaptations necessary for ethnic minority populations; implementation considerations for rural, school-based settings; and evaluation indicators to assess implementation and outcomes. Component specification involved iterative refinement to ensure internal consistency, comprehensiveness, and practical feasibility.

### 3.5 Implementation strategy development

Implementation strategies were developed drawing on principles from implementation science frameworks, particularly the Exploration, Preparation, Implementation, Sustainment (EPIS) framework (25) and systematic compilations of implementation strategies for school mental health contexts (27). Implementation strategies were organized into three phases corresponding to key stages of program adoption: Preparation and Planning, Pilot Implementation, and Full Implementation and Sustainability. For each implementation phase, specific strategies were identified addressing: stakeholder engagement and community participation; needs assessment and cultural adaptation; capacity building and training; resource development; quality assurance and fidelity monitoring; and sustainability planning. Implementation strategies were designed to be pragmatic and feasible within rural school contexts while maintaining fidelity to evidence-based practices.

### 3.6 Framework validation and refinement

While this manuscript presents the initial framework development, ongoing validation and refinement will occur through multiple mechanisms. Planned validation activities include: expert review by psychiatric nursing specialists, child mental health researchers, cultural competence experts, and implementation scientists; community stakeholder consultation with ethnic minority community members, school administrators, teachers, and family caregivers; pilot testing in selected rural school settings; and iterative refinement based on implementation data and stakeholder feedback. This iterative approach to framework development and refinement aligns with best practices in implementation science, which emphasize the importance of continuous quality improvement and adaptation to local contexts while maintaining core evidence-based components (25).

## 4 Theoretical framework

The proposed family-centered psychiatric nursing framework is grounded in three complementary theoretical perspectives that provide comprehensive understanding of the complex factors affecting left-behind children in ethnic minority concentrated areas.

### 4.1 Family stress model

The Family Stress Model, originally developed by Conger et al., (14), provides a theoretical foundation for understanding how economic hardship and parental absence affect family functioning and child outcomes. Originally developed to examine the effects of economic depression on families, this model has been adapted to understand the impacts of parental migration on left-behind children. The model proposes that economic pressure and family disruption create stress that affects family relationships, parenting practices, and ultimately child adjustment (28).

Research applying the Family Stress Model to Chinese left-behind children demonstrates that parental migration creates multiple stressors including: (1) economic uncertainty despite remittances; (2) changes in family structure and role responsibilities; (3) emotional strain related to separation and communication challenges; and (4) social stigma associated with family disruption. These stressors interact to influence child outcomes through their effects on family functioning, caregiver mental health, and parent–child relationships (29). Studies examining the Family Stress Model in Chinese contexts confirm that family economic hardship significantly affects child development through disrupted family processes, with research showing that economic pressure increases stress related to meeting basic family needs and financial obligations (30).

The Family Stress Model emphasizes the importance of family resilience factors that can buffer the negative effects of stress. These resilience factors include strong family communication, effective problem-solving skills, social support networks, and cultural resources that provide meaning and coping strategies. Interventions based on this model focus on strengthening these protective factors while addressing specific stressors that families face (31).

### 4.2 Cultural competence theory

Cultural Competence Theory, as developed by Sue and colleagues, provides a framework for understanding how cultural factors influence mental health, help-seeking behaviors, and treatment effectiveness (15). This theory emphasizes that culture profoundly shapes individuals’ understanding of mental health, acceptable intervention strategies, and preferred sources of help and support. Effective mental health interventions must be adapted to align with cultural values, beliefs, and practices while maintaining evidence-based effectiveness (32).

For ethnic minority populations, cultural competence involves understanding: (1) traditional beliefs about mental health and illness; (2) family structures and decision-making processes; (3) communication patterns and help-seeking behaviors; (4) historical experiences with formal health care systems; and (5) community resources and support systems. Cultural competence also requires recognition of within-group diversity and avoiding stereotyping based on ethnic category alone (33). Research demonstrates that ethnic minority clients’ perceptions of their counselors’ multicultural counseling competence partially mediate the relationship between cultural competence and treatment outcomes (32).

Research examining cultural competence in mental health care for Chinese ethnic minority populations identifies several key cultural factors: emphasis on family harmony and collective well-being; importance of face-saving and avoiding shame; respect for authority and hierarchical relationships; preference for indirect communication styles; and integration of traditional and modern healing approaches. Interventions that acknowledge and incorporate these cultural factors demonstrate greater effectiveness and acceptability (34).

### 4.3 Ecological systems theory

Ecological Systems Theory, developed by Urie Bronfenbrenner, provides a comprehensive framework for understanding the multiple levels of influence on child development and mental health (16). This theory recognizes that children are embedded within nested systems including family, school, community, and broader cultural contexts. Each system level influences child outcomes directly and through interactions with other system levels.

For left-behind children in ethnic minority concentrated areas, ecological factors include: (1) microsystem influences such as family structure, caregiver relationships, and peer interactions; (2) mesosystem interactions between family, school, and community; (3) exosystem factors such as parental work conditions, community economic conditions, and educational policies; and (4) macrosystem influences including cultural values, government policies, and societal attitudes toward ethnic minorities (35). The model highlights the importance of environmental factors, personal characteristics, and contextual factors in shaping development.

The ecological perspective emphasizes that effective interventions must address multiple system levels simultaneously. Individual-focused interventions alone are insufficient to address the complex, multi-level challenges faced by left-behind children (36). Comprehensive interventions must include family-level strategies to strengthen caregiving relationships, school-level approaches to improve educational and mental health supports, community-level initiatives to build social capital and resources, and policy-level advocacy to address structural inequities. Bronfenbrenner later expanded his theory into the bioecological model, emphasizing that proximal processes - the ongoing interactions between a person and their environment - are the primary engines of development (37).

## 5 Proposed framework

### 5.1 Framework overview

The Family-Centered Mental Health Care Framework for Left-Behind Children in Chinese Ethnic Minority Concentrated Areas represents a comprehensive, multi-level intervention model designed specifically for implementation within rural educational settings. This framework integrates psychiatric nursing expertise with cultural competence principles, family-centered care approaches, and school-based service delivery models to address the unique needs of this vulnerable population.

**Figure 1:**
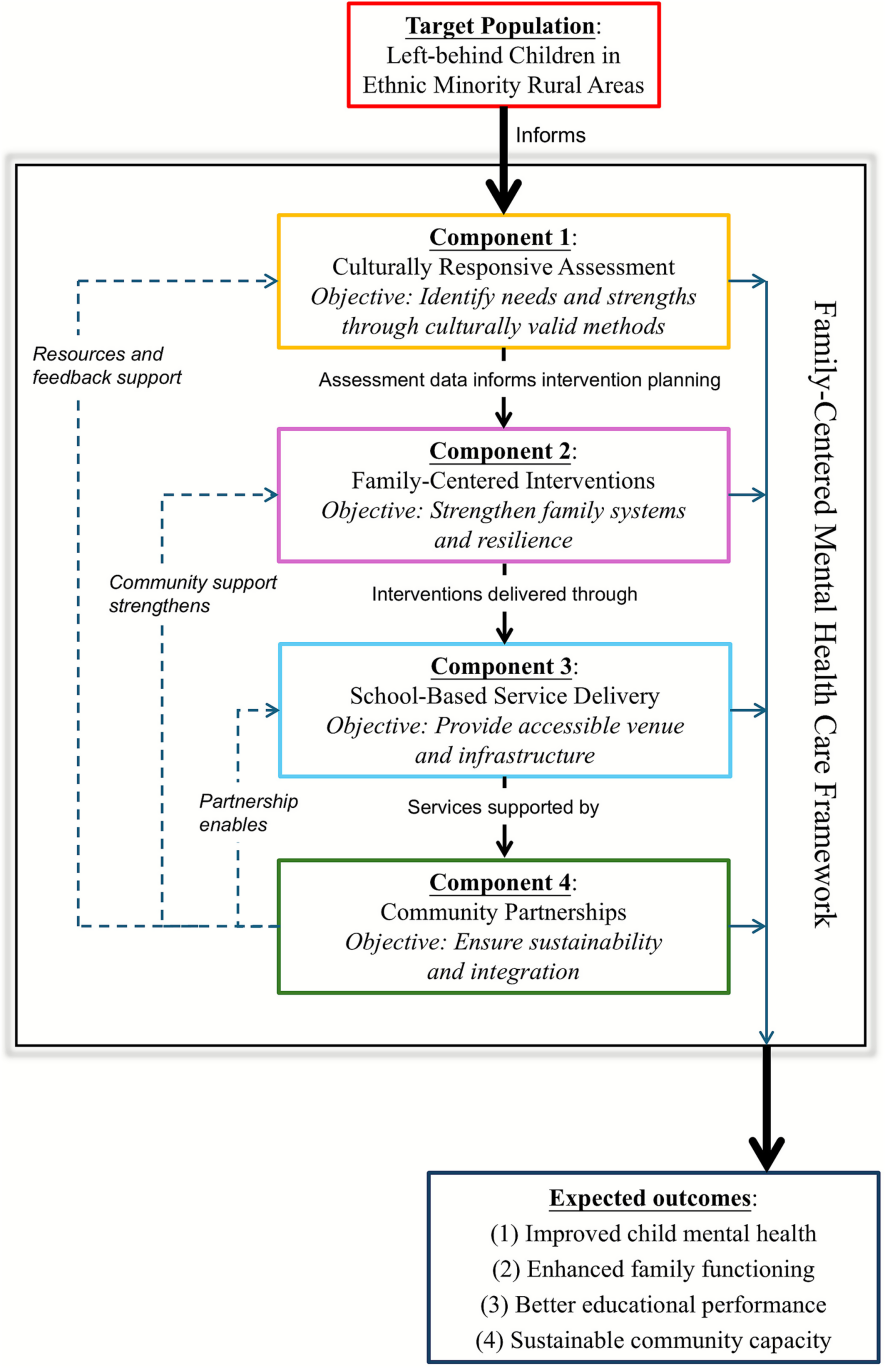
Conceptual model of the family-centered mental health care framework for left-behind children in ethnic minority rural areas.

The framework is structured around four core components that address different levels of intervention while functioning as an integrated system (please see Figure 1 below): (1) Culturally Responsive Assessment Protocols establish the foundation by identifying needs and strengths through culturally valid methods; (2) Family-Centered Intervention Strategies address the identified needs by strengthening family systems and resilience; (3) School-Based Mental Health Service Delivery provides the accessible venue and infrastructure for service implementation; and (4) Community Partnership Models ensure sustainability and integration with broader support systems. Each component incorporates specific strategies, tools, and processes designed to ensure cultural appropriateness, evidence-based effectiveness, and sustainable implementation. These components work synergistically through shared principles of cultural competence, trauma-informed care, and family-centeredness, with psychiatric nurses serving as integrators across all levels.

### 5.2 Component 1: culturally responsive assessment protocols

The first component focuses on developing and implementing assessment protocols that are culturally appropriate, linguistically accessible, and sensitive to the unique experiences of left-behind children in ethnic minority communities. Traditional mental health assessment tools often lack cultural validity and may misinterpret cultural expressions of distress or resilience.

#### 5.2.1 Cultural mental health assessment framework

This framework incorporates multiple assessment domains including: (1) cultural identity and acculturation patterns; (2) family structure and caregiving arrangements; (3) migration history and separation experiences; (4) cultural expressions of psychological distress; (5) traditional healing practices and beliefs; (6) social support networks and community connections; and (7) cultural strengths and resources.

#### 5.2.2 Adapted screening instruments

Existing validated screening tools are culturally adapted for use with ethnic minority left-behind children. This includes translation into local languages, modification of items to reflect cultural concepts, and establishment of culturally appropriate cut-off scores. Examples include adaptations of the Center for Epidemiological Studies Depression Scale, the Pediatric Anxiety Rating Scale, and behavioral screening checklists.

#### 5.2.3 Multi-informant assessment approach

Assessment protocols incorporate multiple perspectives including child self-report, caregiver observations, teacher reports, and peer evaluations. This approach recognizes that cultural factors may influence reporting patterns and that different informants provide unique insights into child functioning across various contexts.

#### 5.2.4 Trauma-informed assessment

Given the potential trauma associated with parental separation and other adverse experiences, assessment protocols incorporate trauma-informed principles. This includes screening for potentially traumatic events, assessing trauma symptoms using culturally appropriate measures, and recognizing cultural factors that may influence trauma expression and recovery.

### 5.3 Component 2: family-centered intervention strategies

The second component focuses on intervention strategies that engage and support the entire family system while recognizing the unique challenges faced by left-behind children and their caregivers. These interventions are designed to strengthen family resilience, improve communication, and enhance caregiving capacity.

#### 5.3.1 Multi-generational family therapy

This intervention model recognizes that many left-behind children are cared for by grandparents or other extended family members. The approach includes psychoeducation about child development and mental health, communication skills training, and strategies for managing intergenerational conflicts. Special emphasis is placed on helping older caregivers understand contemporary stressors while respecting traditional values and practices.

#### 5.3.2 Family communication enhancement

Interventions focus on improving communication between left-behind children and their migrant parents through technology-mediated approaches. This includes training families in effective use of video calling, social media, and other communication technologies, as well as developing structured communication protocols that maintain emotional connections despite physical separation.

#### 5.3.3 Cultural identity development

Interventions help children develop positive ethnic identity while navigating acculturation challenges. This includes activities that celebrate cultural heritage, storytelling traditions that connect children to their cultural roots, and mentorship programs that connect children with positive ethnic minority role models.

#### 5.3.4 Economic literacy and planning

Given the economic factors underlying parental migration, interventions include age-appropriate financial literacy education and family economic planning assistance. This helps families make informed decisions about migration patterns while considering impacts on children’s well-being.

#### 5.3.5 Caregiver support groups

Peer support groups for caregivers provide opportunities for sharing experiences, learning from others, and reducing social isolation. Groups are facilitated by psychiatric nurses with cultural competence training and include structured programming on topics such as child development, mental health awareness, and effective parenting strategies.

### 5.4 Component 3: school-based mental health service delivery

The third component focuses on integrating mental health services within rural educational settings, recognizing schools as primary community institutions with direct access to children and families. This component emphasizes building school capacity for mental health service delivery while maintaining focus on educational outcomes.

#### 5.4.1 Integrated service delivery model

Mental health services are integrated within existing school programming rather than operating as separate, stigmatized services. This includes incorporation of social–emotional learning curricula, integration of mental health screening within routine school health assessments, and embedding mental health promotion activities within regular classroom instruction.

#### 5.4.2 Teacher and staff training

Comprehensive training programs prepare educators to recognize mental health needs, provide appropriate referrals, and support children with mental health challenges within educational settings. Training includes cultural competence components that help staff understand unique challenges faced by ethnic minority left-behind children.

#### 5.4.3 Peer support programs

School-based peer support programs connect left-behind children with trained peer mentors who have successfully navigated similar challenges. These programs provide both emotional support and practical assistance while reducing stigma associated with help-seeking.

#### 5.4.4 Family engagement initiatives

School-based programming includes specific initiatives to engage caregivers and families in children’s education and mental health care. This includes flexible scheduling to accommodate working caregivers, culturally appropriate communication strategies, and family-centered school activities that build community connections.

#### 5.4.5 Crisis response protocols

Schools develop and implement crisis response protocols specifically designed for left-behind children who may be at elevated risk for mental health crises. These protocols include procedures for risk assessment, crisis intervention, family notification, and coordination with mental health professionals.

### 5.5 Component 4: community partnership models

The fourth component focuses on building partnerships between schools, health care systems, community organizations, and cultural institutions to create comprehensive support networks for left-behind children and their families. This component recognizes that sustainable change requires community-wide engagement and resource coordination.

#### 5.5.1 Health care system partnerships

Formal partnerships with local health care providers ensure coordinated care for children with mental health needs. This includes referral protocols, shared care planning, medication management coordination, and joint training initiatives for health care and educational personnel.

#### 5.5.2 Cultural institution engagement

Partnerships with cultural and religious institutions leverage existing community resources and leadership structures. This includes collaboration with traditional healers, religious leaders, and cultural organizations to provide culturally integrated support services.

#### 5.5.3 Community advisory boards

Community advisory boards include representation from ethnic minority communities, parents of left-behind children, educators, health care providers, and community leaders. These boards provide ongoing guidance for framework implementation, cultural adaptation, and sustainability planning.

#### 5.5.4 Resource development and coordination

Community partnerships facilitate development and coordination of resources needed to support left-behind children and families. This includes establishment of emergency assistance funds, coordination of transportation services, development of childcare resources, and creation of recreational and cultural programming.

#### 5.5.5 Advocacy and policy development

Community partnerships engage in advocacy efforts to address policy barriers and promote supportive policies for left-behind children and ethnic minority families. This includes advocacy for educational equity, mental health parity, immigration policy reform, and rural development initiatives.

### 5.6 Implementation team training and capacity building

#### 5.6.1 Core implementation team and training requirements

The implementation team includes: psychiatric nurses (primary mental health specialists), school mental health coordinators (service integration facilitators), teachers and educational staff (frontline identifiers), community health workers and cultural liaisons (cultural brokers), and family caregivers (care partners). Each role requires specific foundational and specialized training. Foundational training (16–24 h for all team members) covers: framework overview; understanding left-behind children in ethnic minority contexts; mental health literacy; cultural competence principles; trauma-informed care; family-centered care philosophy; and communication skills.

Role-specific advanced training includes: psychiatric nurses (40–60 h): culturally adapted assessment, evidence-based interventions, family therapy, school consultation, crisis intervention, and supervision skills; school coordinators (24–32 h): care coordination, referral processes, data management, and team facilitation; teachers (8–12 h): recognizing mental health concerns, trauma-sensitive classroom management, and referral processes; community health workers (16–24 h): community engagement, cultural brokering, family navigation, and interdisciplinary collaboration.

#### 5.6.2 Training delivery and competency assessment

Training employs multiple methods: didactic instruction, experiential learning (role-plays, simulations), case-based learning, cultural immersion, mentorship, and technology-enhanced learning. All materials are culturally adapted and available in relevant languages. Competency assessment includes: knowledge assessments, skills demonstrations using structured checklists, case presentations, supervisor evaluation, and portfolio development. Minimum competency standards are established for each role before independent practice.

#### 5.6.3 Ongoing development, supervision, and quality monitoring

Continuous learning includes: weekly clinical supervision for psychiatric nurses (1–2 h), regular supervision for all team members, bi-weekly group supervision, annual continuing education (minimum 20 h for clinical staff, 10 h for support staff), communities of practice, and performance feedback with coaching. Quality monitoring ensures fidelity through: structured fidelity assessments (quarterly), continuous monitoring of quality indicators (utilization rates, completion of assessments, timeliness, satisfaction, outcomes), and regular data review for continuous improvement. Staff well-being support includes stress assessment, peer support groups, access to mental health services, manageable workloads, debriefing protocols, and work-life balance promotion.

## 6 Implementation strategies

### 6.1 Phase 1: preparation and planning

The initial phase focuses on comprehensive preparation and planning to ensure the framework is tailored to the specific community context, with primary emphasis on establishing foundational capacity for Component 1 (Assessment Protocols) and Component 4 (Community Partnerships). This process begins with systematic stakeholder engagement, bringing together school administrators, teachers, parents, and community leaders through forums and focus groups to ensure the implementation plan reflects local needs and priorities. A thorough needs assessment is then conducted to identify existing services, resource gaps, and community strengths. The insights gathered directly inform the crucial step of cultural adaptation, where framework components - particularly assessment tools and screening instruments (Component 1) - are refined in consultation with cultural experts and pilot-tested with community members to ensure their relevance and appropriateness.

Concurrent with this community-focused work, the second part of this phase concentrates on building the internal capacity required for successful delivery. This involves the targeted recruitment and specialized training of qualified psychiatric nurses and other mental health professionals with experience in rural and culturally competent practice. Training programs cover framework-specific content, trauma-informed care, and collaborative skills necessary for delivering all framework components. In parallel, the culturally adapted assessment instruments (Component 1), intervention protocols (Component 2), training materials, and community partnership agreements (Component 4) are fully developed and finalized. This foundational phase concludes when a competent implementation team is in place, culturally adapted assessment tools are validated, community partnerships are formalized, and all necessary resources are ready for pilot implementation.

### 6.2 Phase 2: pilot implementation

The second phase involves pilot implementation in carefully selected schools and communities to test framework components systematically, refine implementation processes, and demonstrate feasibility and effectiveness. Pilot sites are selected based on criteria including community readiness, administrative support, available resources, and population characteristics, ensuring representation of diverse ethnic minority communities and varying rural contexts.

Framework components are implemented in a deliberately sequenced manner to allow for systematic testing and refinement. Implementation begins with Component 1 (Culturally Responsive Assessment Protocols), establishing systematic screening and assessment procedures for identifying children’s mental health needs and family strengths. Once assessment processes are functioning effectively, Component 2 (Family-Centered Intervention Strategies) is introduced, with psychiatric nurses delivering targeted interventions such as family therapy, caregiver support groups, and communication enhancement programs based on assessment findings. Subsequently, Component 3 (School-Based Mental Health Service Delivery) is expanded through teacher training, peer support program development, and integration of mental health promotion activities within school programming. Throughout this sequential implementation, Component 4 (Community Partnership Models) continues to develop and strengthen, with formal collaboration mechanisms established between schools, health care providers, and cultural institutions.

Pilot implementation incorporates continuous quality improvement processes including regular data collection, stakeholder feedback, and program refinement to ensure that implementation challenges are identified and addressed promptly. Throughout this phase, ongoing training and technical assistance support pilot site staff through regular consultation, skills development workshops, problem-solving assistance, and peer learning opportunities. Comprehensive documentation of implementation processes, challenges, and outcomes occurs alongside evaluation activities that examine both process indicators (assessment completion rates, intervention participation, service utilization patterns, stakeholder satisfaction) and outcome measures (child mental health status, family functioning, educational performance). This systematic approach to pilot testing allows for iterative refinement of all framework components while building evidence for effectiveness and generating lessons learned that inform subsequent scaling efforts.

### 6.3 Phase 3: full implementation and sustainability

The final phase involves full framework implementation across target communities with all four components operating in an integrated, mutually reinforcing manner, alongside development of comprehensive sustainability strategies to ensure long-term program continuation. Based on pilot results, framework implementation is scaled to additional schools and communities through processes that incorporate lessons learned from pilot experiences and include systematic planning for resource allocation, staff development, and quality assurance.

Full implementation includes establishment of quality assurance systems to ensure consistent, high-quality service delivery across all framework components and implementation sites through staff certification programs, service delivery standards, regular fidelity monitoring procedures, and outcome monitoring systems that maintain adherence to the framework while allowing for necessary local adaptations. Component 1 assessment protocols become integrated within routine school health procedures; Component 2 interventions are offered through established referral pathways and service structures; Component 3 school-based services are embedded within educational programming with trained staff; and Component 4 community partnerships function through formalized governance structures and resource-sharing agreements.

Comprehensive sustainability planning addresses funding, staffing, organizational, and political factors necessary for long-term program continuation through diversification of funding sources, integration with existing health and education systems, development of local leadership capacity, and policy advocacy efforts. Successful implementation experiences are systematically documented and disseminated to support replication in other communities through publication of implementation guides, training materials, evaluation reports, and presentations at professional conferences. This phase ensures that the integrated framework becomes embedded within community systems and structures, creating lasting change that extends beyond the initial implementation period while establishing a foundation for broader dissemination and replication efforts.

## 7 Evaluation framework

### 7.1 Process evaluation

Process evaluation examines implementation fidelity, service delivery quality, and stakeholder satisfaction to ensure that framework components are implemented as intended and meet community needs through comprehensive monitoring and assessment activities. Given the school-based nature of service delivery and the critical importance of educational success for long-term child outcomes, evaluation includes comprehensive examination of educational outcomes such as academic performance across subject areas, school engagement and motivation indicators, attendance rates, disciplinary incidents, and teacher-reported behavioral improvements in classroom settings. Fidelity monitoring is critical for this multi-component intervention. It ensures that core principles are maintained while allowing for necessary cultural adaptations for diverse ethnic minority populations.

Service utilization analysis provides crucial insights into program reach and accessibility by examining who is receiving services, what types of services are being provided, and how frequently services are utilized across different demographic groups and geographic areas within the target communities. This analysis helps identify potential access barriers, service gaps, and disparities in utilization patterns that may reflect cultural, linguistic, or systemic obstacles to care, informing continuous service improvement efforts and ensuring equitable access across all ethnic minority subgroups. Concurrent stakeholder satisfaction surveys of children, families, school staff, and community members examine satisfaction with services, perceived helpfulness of interventions, cultural appropriateness of service delivery, and suggestions for improvement, providing essential feedback that guides ongoing program refinement and quality improvement efforts.

Staff competence assessment ensures that service providers maintain the necessary knowledge, skills, and cultural competence required for effective framework implementation through regular review of training completion, supervision documentation, and direct assessment of clinical skills and cultural responsiveness. This ongoing professional development monitoring is particularly important given the specialized nature of working with left-behind children in ethnic minority communities, requiring providers to demonstrate proficiency in trauma-informed care, cultural competence, family systems intervention, and collaborative care models that integrate mental health services within educational settings.

### 7.2 Outcome evaluation

Outcome evaluation examines the effectiveness of the framework in improving mental health outcomes for left-behind children and their families through comprehensive assessment of individual, family, educational, and community-level changes. Primary child mental health outcomes include standardized assessments of depression, anxiety, behavioral problems, and overall psychological well-being using culturally adapted instruments that have demonstrated validity and reliability with Chinese ethnic minority populations, with outcome assessments conducted at baseline, regular intervals during service provision, and multiple follow-up periods after service completion to capture both immediate and sustained intervention effects. Family-level outcomes encompass measures of family communication patterns, caregiver mental health and stress levels, family resilience and coping strategies, and parent–child relationships quality, examining the framework’s effectiveness in strengthening family systems and support networks while addressing the unique challenges faced by families experiencing parental migration and cultural adaptation pressures.

Because the framework is school-based, educational outcomes are a critical measure of success. The evaluation will track academic performance, school engagement, attendance, disciplinary incidents, and teacher-reported classroom behavior. These metrics indicate the framework’s impact on the intersection of mental health and educational functioning. Community-level outcomes examine broader impacts including changes in mental health awareness and literacy, help-seeking behaviors and attitudes toward mental health services, stigma reduction around mental health issues, and overall community capacity for addressing mental health needs through enhanced collaboration between schools, health care providers, and cultural institutions.

Long-term follow-up studies examine the sustainability of intervention effects over extended periods and identify individual, family, and community factors associated with positive long-term outcomes, providing critical evidence for understanding the developmental trajectories of left-behind children who receive services and informing strategies for maintaining intervention gains over time. These longitudinal evaluations are particularly important for understanding how early intervention during childhood influences adolescent and young adult outcomes, including academic achievement, mental health stability, family relationship quality, and social functioning within ethnic minority communities. The evaluation framework also incorporates cost-effectiveness analysis to examine the economic benefits of the intervention relative to costs, providing essential information for sustainability planning and policy advocacy efforts aimed at securing long-term funding and support for comprehensive mental health programming in rural ethnic minority communities.

## 8 Discussion

### 8.1 Framework advantages and innovation

The proposed Family-Centered Mental Health Care Framework represents a significant innovation in addressing mental health needs of left-behind children in ethnic minority concentrated areas, distinguished by several key features that offer particular advantages for this vulnerable population. Unlike generic mental health interventions that are later adapted for cultural populations, this framework is designed from the ground up with cultural competence as a central organizing principle, ensuring that every component incorporates cultural factors as essential elements rather than add-on considerations. This approach ensures greater cultural authenticity and effectiveness while avoiding the limitations of superficial cultural adaptations that often fail to address deep-rooted cultural beliefs, communication patterns, and help-seeking behaviors. The framework’s emphasis on family-centered care addresses the reality that left-behind children are embedded within complex family systems that extend beyond nuclear family boundaries, with the multi-generational approach recognizing that effective interventions must engage grandparents, extended family members, and absent parents as integral participants in children’s mental health care, reflecting the collectivistic nature of Chinese ethnic minority cultures where family harmony and intergenerational relationships are paramount.

The framework’s integration of mental health services within schools is a key innovation. This school-based model addresses systemic barriers by leveraging existing infrastructure and community trust. Situating services in schools reduces stigma and overcomes practical barriers like transportation and scheduling that typically hinder access for rural ethnic minority families. As trusted community anchors, schools provide crucial opportunities for early identification, prevention, and intervention that would otherwise be missed. The framework’s emphasis on community partnerships leverages existing resources and leadership structures while building sustainable community capacity for ongoing service delivery, an approach that is particularly crucial in rural areas where external resources may be limited and community buy-in is essential for program success and long-term sustainability.

### 8.2 Implementation challenges and solutions

Implementing the proposed framework will require proactive planning to address several significant challenges unique to rural ethnic minority communities. A critical obstacle is the severe shortage of qualified mental health professionals, which can be addressed through specialized training programs, financial incentives, and technology-mediated supervision to build local capacity. Furthermore, the framework’s comprehensive nature requires substantial resources that are often scarce in rural areas, necessitating innovative funding strategies that blend sources from education, health care, and social services, with cost-effectiveness demonstrated through rigorous evaluation. Finally, implementation must navigate significant cultural and geographic barriers by developing flexible adaptation protocols in partnership with local cultural leaders and by utilizing mobile service delivery models and technology-mediated interventions to overcome the challenges of geographic isolation.

### 8.3 Implications

#### 8.3.1 Implications for practice

The framework expands psychiatric nursing roles beyond clinic-based care to include school consultation, family systems intervention, community partnership development, care coordination, and prevention programming. Cultural competence must be systematically integrated through understanding specific cultural values and practices, recognizing historical trauma, acknowledging within-group diversity, and leveraging cultural strengths. Organizations must provide cultural competence training, employ culturally diverse staff, and establish cultural consultation structures. Family-centered approaches require shifting from child-focused to family-system interventions that assess family dynamics, engage caregivers as partners, provide caregiver support, address family stressors, and recognize diverse family structures. Interdisciplinary collaboration demands partnerships with educators, primary care providers, community agencies, and cultural organizations, requiring mutual understanding of roles, shared communication systems, collaborative decision-making, and clear coordination protocols. Trauma-informed care requires universal trauma screening, recognizing trauma responses, avoiding re-traumatization, providing evidence-based trauma treatment, and maintaining organizational trauma-informed practices.

#### 8.3.2 Implications for policy

The proposed framework aligns directly with China’s major national policy initiatives and provides a concrete mechanism for achieving their objectives. The framework operationalizes key goals of the Healthy China 2030 initiative by establishing school-based mental health services that promote mental health, prevent disorders, and reduce urban–rural health disparities for vulnerable populations. It supports implementation of the 2013 Mental Health Law of the People’s Republic of China through community-based service models that increase access, promote awareness, and diversify the mental health workforce by emphasizing psychiatric nurse-led care in underserved areas. The framework also advances rural revitalization and poverty alleviation strategies by building local health service capacity, strengthening human capital development, and supporting families in making informed decisions about migration patterns that consider both economic needs and children’s developmental well-being. By integrating mental health services within educational settings and strengthening community partnerships, the framework contributes to comprehensive rural development that addresses not only income but also health, education, and social support dimensions of poverty.

Successful framework implementation and sustainability require supportive policies across multiple domains. Educational policies must mandate comprehensive school mental health programming with dedicated funding, adequate staffing ratios, required mental health training for educators, and formal school-health care collaboration mechanisms. Health care policies should expand psychiatric nursing scope of practice for school-based services, ensure equitable reimbursement, provide rural practice incentives, support telehealth infrastructure, and fund workforce development targeting rural and ethnic minority contexts. System integration policies must facilitate formal health-education partnerships through shared funding mechanisms, aligned accountability metrics, coordinated data systems with privacy protections, and joint governance structures. Cultural competence policies should ensure ethnic minority community participation in policy development, fund culturally specific services and adaptation research, mandate cultural competence training for all professionals serving ethnic minority populations, require disaggregated data collection to monitor disparities, and guarantee language access through interpretation services and translated materials. These policy recommendations create an enabling environment for framework adoption while advancing broader goals of health equity, rural development, and social inclusion for ethnic minority populations.

#### 8.3.3 Implications for research

Effectiveness research must establish whether the framework improves mental health, family functioning, and educational outcomes through RCTs, quasi-experimental designs, longitudinal studies, and mixed-methods approaches. Cultural adaptation research should identify critical adaptations for different ethnic groups, determine how to maintain fidelity while adapting, and examine cultural strengths’ role in resilience, using community-based participatory approaches. Implementation research must identify factors supporting adoption and sustainability, determine effective implementation strategies, examine adaptation for different contexts, assess cost-effectiveness, and explore technology’s role. Health equity research must examine whether the framework reduces disparities in access, quality, and outcomes, requiring disaggregated data and attention to structural factors. Technology research should explore telehealth models, technology-mediated family engagement, digital interventions for rural populations, and strategies ensuring equitable technology access.

#### 8.3.4 Implications for education

Psychiatric nursing curricula must include comprehensive child/adolescent mental health content, family-centered approaches, cultural competence, trauma-informed care, community mental health, collaborative care models, school-based delivery, and rural health practice. Advanced practice education should add advanced psychotherapy, population health, program development, health policy, leadership, consultation skills, and clinical supervision. Continuing professional development priorities include evidence-based practice training, cultural competence, trauma-informed care, telehealth skills, and collaboration competencies, delivered through accessible formats for rural practitioners. Interdisciplinary education should provide shared learning experiences across nursing, education, social work, psychology, and counseling programs. Family and community education includes mental health literacy, parenting programs, school staff training, community health worker preparation, and leadership development for ethnic minority advocates. Faculty development requires recruiting diverse faculty, providing professional development in rural health and cultural competence, creating academic-community partnerships, and supporting faculty scholarship in child mental health and health equity.

### 8.4 Limitations and future directions

While the proposed framework offers significant theoretical and practical advantages, several important limitations must be acknowledged. The framework is based primarily on existing literature, and evidence specifically examining this population remains limited. Future research, including rigorous evaluation studies, is needed to strengthen the evidence base and empirically refine the framework’s components. The framework’s design for Chinese ethnic minority areas raises questions about its generalizability. Future work should involve careful examination of adaptation strategies for other cultural contexts to determine which framework elements are universal versus culturally specific. Furthermore, the comprehensive nature of the framework may require substantial human and financial resources that exceed the capacity of some rural communities. This necessitates the development of scaled implementation models and innovative financing mechanisms to support sustainable service delivery. Long-term sustainability in resource-limited areas remains a significant challenge, requiring ongoing research into community capacity building and supportive policy advocacy to ensure continued service availability.

## 9 Conclusion

Left-behind children in Chinese ethnic minority concentrated areas face profound mental health challenges at the intersection of parental migration, rural isolation, cultural marginalization, and limited mental health resources. This framework addresses this critical gap by proposing a comprehensive, culturally responsive psychiatric nursing model that delivers family-centered care within school-based settings. The framework’s distinguishing features - cultural specificity designed from the ground up rather than adapted from generic models, multi-generational family engagement, school-based accessibility that reduces stigma and overcomes barriers, community partnerships that build sustainable capacity, and psychiatric nurse leadership in integrated care - work synergistically to address the multi-level factors affecting these children’s mental health and development. Successful implementation requires multi-level commitment from practitioners developing specialized competencies, organizations providing resources and collaboration mechanisms, policymakers enacting supportive policies, researchers generating rigorous evidence, and educators preparing the workforce. While designed for Chinese contexts, this framework’s core principles offer a replicable model for addressing vulnerable children’s mental health globally wherever parental migration, rural isolation, and service gaps create disparities. Ultimately, this work demonstrates that equitable, culturally responsive mental health care for underserved populations is achievable through systematic, theory-informed, and community-engaged approaches that position psychiatric nursing at the forefront of integrated service delivery.

## Data Availability

The original contributions presented in the study are included in the article/supplementary material, further inquiries can be directed to the corresponding author.

## References

[1] National Bureau of Statistics, UNICEF, and United Nations Population Fund. (2023). Child population in China 2020: facts and data. Available online at: https://www.unicef.cn/media/24496/file/2020%E5%B9%B4%E4%B8%AD%E5%9B%BD%E5%84%BF%E7%AB%A5%E4%BA%BA%E5%8F%A3%E7%8A%B6%E5%86%B5%E4%BA%8B%E5%AE%9E%E4%B8%8E%E6%95%B0%E6%8D%AE.pdf

[2] Chen Y Li Y Zeng J. Parental migration patterns and children depression in China’s ethnic minority rural areas: a latent profile analysis. Acta Psychol. (2025) 254:104836. doi: 10.1016/j.actpsy.2025.104836, 39983425

[3] Zhang J. Mental health education in small rural schools in ethnic frontier regions. Soc Sci. (2023) 315:140–6.

[4] Zhang X Peng M. Psychological and behavioral issues among left-behind children in ethnic regions of Sichuan and their impact on learning difficulties. Education Science Forum. (2021) 23:31–4.

[5] Morales DA Barksdale CL Beckel-Mitchener AC. A call to action to address rural mental health disparities. J Clinical Transl Sci. (2020) 4:463–7. doi: 10.1017/cts.2020.42, 33244437 PMC7681156

[6] McGuire TG Miranda J. New evidence regarding racial and ethnic disparities in mental health: policy implications. Health Aff. (2008) 27:393–403. doi: 10.1377/hlthaff.27.2.393, 18332495 PMC3928067

[7] Hu M Li X Zhu Y Chen Z Lai C Liu R . The role of family caregiving in the management of individuals with mental illnesses and the outcome of family-based interventions for mental illnesses in China: a scoping review. Lancet Regional Health. (2025) 56:101184. doi: 10.1016/j.lanwpc.2024.101184, 40226781 PMC11992586

[8] Phillips R Durkin M Engward H Cable G Iancu M. The impact of caring for family members with mental illnesses on the caregiver: a scoping review. Health Promot Int. (2023) 38:daac049. doi: 10.1093/heapro/daac049, 35472137 PMC10269136

[9] Barnes MD Hanson CL Novilla LB Magnusson BM Crandall AC Bradford G. Family-centered health promotion: perspectives for engaging families and achieving better health outcomes. Inquiry. (2020) 57:0046958020923537. doi: 10.1177/0046958020923537, 32500768 PMC7278332

[10] Hoover S Bostic J. Schools as a vital component of the child and adolescent mental health system. Psychiatr Serv. (2021) 72:37–48. doi: 10.1176/appi.ps.201900575, 33138711

[11] Stephan SH Sugai G Lever N Connors E. Strategies for integrating mental health into schools via a multitiered system of support. Child Adolesc Psychiatr Clin N Am. (2015) 24:211–31. doi: 10.1016/j.chc.2014.12.002, 25773320

[12] Moore S Timpe Z Rasberry CN Hertz M Verlenden J Spencer P . Disparities in the implementation of school-based mental health supports among K–12 public schools. Psychiatr Serv. (2024) 75:17–24. doi: 10.1176/appi.ps.20220558, 37312505 PMC10719411

[13] Delaney KR Burke P DeSocio J Greenberg CS Sharp D. Building mental health and caring for vulnerable children: increasing prevention, access, and equity. Nurs Outlook. (2018) 66:590–3. doi: 10.1016/j.outlook.2018.10.004, 30502886

[14] Conger R. D. Elder G. H. Jr. Lorenz F. O. Simons R. L. Whitbeck L. B. (1994). Families in troubled times: Adapting to change in rural America. 11–303. New York, NY: Aldine de Gruyter.

[15] Sue S. In search of cultural competence in psychotherapy and counseling. Am Psychol. (1998) 53:440–8. doi: 10.1037/0003-066X.53.4.440, 9572007

[16] Bronfenbrenner U. Ecological systems theory In. Kazdin AE (ed). Encyclopedia of psychology, Vol. 3. Washington, DC: American Psychological Association (2000). 129–33.

[17] Mao M Zang L Zhang H. The effects of parental absence on children development: evidence from left-behind children in China. Int J Environ Res Public Health. (2020) 17:6770. doi: 10.3390/ijerph17186770, 32957472 PMC7559575

[18] Hu H Gao J Jiang H Jiang H Guo S Chen K . A comparative study of behavior problems among left-behind children, migrant children and local children. Int J Environ Res Public Health. (2018) 15:655. doi: 10.3390/ijerph15040655, 29614783 PMC5923697

[19] Huang L Zhang S Bian B Zhou M Bi Z. Peer effects of depression between left-behind and non-left-behind children: quasi-experimental evidence from rural China. Child Adolesc Psychiatry Ment Health. (2023) 17:72. doi: 10.1186/s13034-023-00602-1, 37308963 PMC10262428

[20] Xiong Y Li X Li H Qu C Liu M Lu C . A meta-analysis of loneliness among left-behind children in China. Curr Psychol. (2024) 43:10660–8. doi: 10.1007/s12144-023-04882-w

[21] Weinbrecht A Rieckmann N Renneberg B. Acceptance and efficacy of interventions for family caregivers of elderly persons with a mental disorder: a meta-analysis. Int Psychogeriatr. (2016) 28:1615–29. doi: 10.1017/S1041610216000806, 27268305

[22] Liu H Liu L Jin X. The impact of parental remote migration and parent-child relation types on the psychological resilience of rural left-behind children in China. Int J Environ Res Public Health. (2020) 17:5388. doi: 10.3390/ijerph17155388, 32726979 PMC7432675

[23] Zhou Y Zheng M He Y Zhang J Guo T Wang Q . Impact of family environment in rural China on loneliness, depression, and internet addiction among children and adolescents. Eur J Investigation Health Psychol Educ. (2025) 15:68. doi: 10.3390/ejihpe15050068, 40422297 PMC12110286

[24] Jin F Liu Z Liu Y Yao C Cheng Y Peking University First Hospital, Beijing, China . Health status of left-behind children and parenting behaviors of caregivers in poor rural areas—6 provinces, China, 2018. China CDC Wkly. (2021) 3:54–7. doi: 10.46234/ccdcw2021.017, 34594956 PMC8392933

[25] Lyon AR Connors EH Lawson GM Nadeem E Owens JS. Implementation science in school mental health: a 10-year Progress update and development of a new research agenda. Sch Ment Heal. (2024) 16:1013–37. doi: 10.1007/s12310-024-09731-0, 40822252 PMC12352494

[26] Ti-enkawol Nachinab G Armstrong SJ. The development of a framework for clinical education programme of undergraduate nursing students in Ghana. BMC Nurs. (2024) 23:263. doi: 10.1186/s12912-024-01915-y, 38654226 PMC11036577

[27] Lawson GM Azad G. School-based mental health interventions: recommendations for selecting and reporting implementation strategies. J Sch Health. (2024) 94:581–5. doi: 10.1111/josh.13458, 38627895 PMC11618845

[28] Conger RD Conger KJ Elder GH Lorenz FO Simons RL Whitbeck LB. A family process model of economic hardship and adjustment of early adolescent boys. Child Dev. (1992) 63:526. doi: 10.2307/1131344 1600820

[29] Zhang X Krishnakumar A Narine L. Family economic hardship and child outcomes: test of family stress model in the Chinese context. J Fam Psychol. (2020) 34:960–8. doi: 10.1037/fam0000670, 32406732

[30] Neppl TK Senia JM Donnellan MB. Effects of economic hardship: testing the family stress model over time. J Fam Psychol. (2016) 30:12–21. doi: 10.1037/fam0000168, 26551658 PMC4742411

[31] Xu Y Wu Q Levkoff SE Jedwab M. Material hardship and parenting stress among grandparent kinship providers during the COVID-19 pandemic: the mediating role of grandparents’ mental health. Child Abuse Negl. (2020) 110:104700. doi: 10.1016/j.chiabu.2020.104700, 32854948 PMC7444952

[32] Sue S Zane N Hall GCN Berger LK. The case for cultural competency in psychotherapeutic interventions. Annu Rev Psychol. (2009) 60:525–48. doi: 10.1146/annurev.psych.60.110707.163651 18729724 PMC2793275

[33] Chu J Leino A Pflum S Sue S. A model for the theoretical basis of cultural competency to guide psychotherapy. Prof Psychol Res Pract. (2016) 47:18–29. doi: 10.1037/pro0000055

[34] Berger LK Zane N Hwang W-C. Therapist ethnicity and treatment orientation differences in multicultural counseling competencies. Asian Am J Psychol. (2014) 5:53–65. doi: 10.1037/a0036178, 25580187 PMC4286210

[35] Bronfenbrenner U Morris PA. The bioecological model of human development. In. Lerner RM Damon W (eds), Handbook of child psychology: Theoretical models of human development, Vol. 1. 6th ed. Hoboken, NJ: John Wiley & Sons, Inc. (2006). 793–828.

[36] Tong P An IS. Review of studies applying Bronfenbrenner’s bioecological theory in international and intercultural education research. Front Psychol. (2024) 14:1233925. doi: 10.3389/fpsyg.2023.1233925, 38259539 PMC10801006

[37] Rosa EM Tudge J. Urie Bronfenbrenner’s theory of human development: its evolution from ecology to bioecology. J Fam Theory Rev. (2013) 5:243–58. doi: 10.1111/jftr.12022

